# Evaluation of delay discounting as a transdiagnostic research domain criteria indicator in 1388 general community adults

**DOI:** 10.1017/S0033291721005110

**Published:** 2023-03

**Authors:** E. E. Levitt, A. Oshri, M. Amlung, L. A. Ray, S. Sanchez-Roige, A. A. Palmer, J. MacKillop

**Affiliations:** 1 Peter Boris Centre for Addictions Research, McMaster University & St. Joseph's Healthcare Hamilton, Hamilton, Ontario, Canada; 2 Homewood Research Institute, Guelph, Ontario, Canada; 3 Department of Human Development and Family Science, Athens, Georgia, United States; 4 Department of Applied Behavioral Science, Cofrin Logan Center for Addiction Research and Treatment, University of Kansas, Lawrence, Kansas, United States; 5 Department of Psychology, University of California, Los Angeles, California, United States; 6 Department of Psychiatry, University of California San Diego, San Diego, California, United States; 7 Institute for Genomic Medicine, University of California San Diego, San Diego, California, United States

**Keywords:** Addiction, delay discounting, psychiatric disorder, RDoC, transdiagnostic

## Abstract

**Background:**

The Research Domain Criteria (RDoC) approach proposes a novel psychiatric nosology using transdiagnostic dimensional mechanistic constructs. One candidate RDoC indicator is delay discounting (DD), a behavioral economic measure of impulsivity, based predominantly on studies examining DD and individual conditions. The current study sought to evaluate the transdiagnostic significance of DD in relation to several psychiatric conditions concurrently.

**Methods:**

Participants were 1388 community adults (18–65) who completed an in-person assessment, including measures of DD, substance use, depression, anxiety, posttraumatic stress disorder, and attention-deficit hyperactivity disorder (ADHD). Relations between DD and psychopathology were examined with three strategies: first, examining differences by individual condition using clinical cut-offs; second, examining DD in relation to latent psychopathology variables via principal components analysis (PCA); and third, examining DD and all psychopathology simultaneously via structural equation modeling (SEM).

**Results:**

Individual analyses revealed elevations in DD were present in participants screening positive for multiple substance use disorders (tobacco, cannabis, and drug use disorder), ADHD, major depressive disorder (MDD), and an anxiety disorder (*p*s < 0.05–0.001). The PCA produced two latent components (substance involvement *v.* the other mental health indicators) and DD was significantly associated with both (*p*s < 0.001). In the SEM, unique significant positive associations were observed between the DD latent variable and tobacco, cannabis, and MDD (*p*s < 0.05–0.001).

**Conclusions:**

These results provide some support for DD as a transdiagnostic indicator, but also suggest that studies of individual syndromes may include confounding via comorbidities. Further systematic investigation of DD as an RDoC indicator is warranted.

## Introduction

Understanding psychiatric illness relies on classifying mental illness into discrete and independent categories using systems such as the Diagnostic and Statistical Manual of Mental Disorders and International Classification of Diseases. However, a fundamental concern with diagnostic categories is that they define disorders exclusively by signs and symptoms associated with the individual's subjective experiences and overt presentations, rather than underlying psychological and neurobiological substrates (Lilienfeld, 2014; Lilienfeld & Treadway, 2016). Furthermore, there is a substantive overlap of symptomology across mental illnesses and significant heterogeneity within disorders. Collectively, these issues adversely impact progress in understanding and diagnosing mental disorders (Etkin & Cuthbert, 2014).

Novel approaches have been developed that challenge the notion of categorical classification systems and one prominent framework is the National Institute of Mental Health's (NIMH) Research Domain Criteria (RDoC) (Insel et al., 2010). Specifically, RDoC seeks to develop a new mental health nosology that focuses on transdiagnostic dimensional constructs that reflect the mechanisms that cause and maintain psychiatric disorders (Kozak & Cuthbert, 2016). These transdiagnostic constructs are anticipated to provide a deeper understanding of psychopathology and to promote the use of higher-resolution dimensional measurements, holding promise of enhancing prevention, detection, and treatment of mental illness (Sharp, Miller, & Heller, 2015). In addition, RDoC seeks to contribute to a shift toward precision medicine, which focuses on characterizing the individual pathophysiological features of a disease in an individual to optimize treatment (Cuthbert & Insel, 2013). In psychiatry, there is typically no single pathognomonic feature of a disorder that yields a diagnosis. As such, RDoC aims to reconceptualize the diagnostic classification system to identify specific psychological and biological indicators that allow for a more objective, accurate, and reliable diagnostic system, one that is more amenable to research (Kelly, Clarke, Cryan, & Dinan, 2018). Importantly, RDoC is not the only novel framework for nosology in psychiatry (e.g. the Hierarchical Taxonomy of Psychopathology; Kotov, Krueger, & Watson, 2018).

One candidate RDoC indicator is delay discounting (DD), a person's orientation toward smaller immediate rewards over larger delayed rewards that are considered a behavioral economic index of impulsivity (Madden & Bickel, 2010). Typically, DD is measured using decision-making tasks, where the value of the reward and the delay in time are systematically varied, or pre-configured choices reflecting time-reward trade-offs of differing discounting rates. The higher the discounting rate of delayed rewards, the more impulsive the individual is considered. DD is highly relevant to RDoC as a candidate transdiagnostic indicator because it has been found to be elevated in numerous psychiatric conditions. Studies have found significantly increased levels of DD in individuals with alcohol use disorder, tobacco use disorder, opioid use disorder, other substance use disorders, and gambling disorder (e.g. Bickel, Odum, & Madden, 1999; MacKillop, Anderson, Castelda, Mattson, & Donovick, 2006; Madden, Petry, Badger, & Bickel, 1997; Petry, 2001). Elevated levels of DD have also been observed in a number of other conditions, including attention-deficit hyperactivity disorder (ADHD) (Barkley, Edwards, Laneri, Fletcher, & Metevia, 2001; Demurie, Roeyers, Baeyens, & Sonuga-Barke, 2012; Hurst, Kepley, McCalla, & Livermore, 2011), obesity and eating disorders (Amlung, Petker, Jackson, Balodis, & MacKillop, 2016; Manwaring, Green, Myerson, Strube, & Wilfley, 2011; Stojek & MacKillop, 2017); and major depressive disorder and anxiety (Pulcu et al., 2014; Steinglass et al., 2017).

Meta-analyses of individual studies likewise implicate DD with multiple forms of psychopathology. Syntheses of investigations of addiction using both case-control (MacKillop et al., 2011) and dimensional designs (Amlung, Vedelago, Acker, Balodis, & MacKillop, 2017) have reported significant associations across studies. Recently, a meta-analysis of DD and psychiatric conditions other than addictive disorders also found consistent evidence of elevated DD across disorders (Amlung et al., 2019). Of particular interest, extremes on the spectrum of DD are differentially associated with disorders characterized by self-regulatory under control *v.* over control. For example, there is consistent evidence of significantly higher DD in disorders characterized by under control, such as substance use disorders (Amlung et al., 2017) and ADHD (Jackson & MacKillop, 2016), and consistent evidence of significantly lower DD in anorexia (Amlung et al., 2019). Although the etiological role of DD remains actively under investigation (e.g. Oshri *et al*., 2019; Owens *et al*., 2017), there is evidence that it is a heritable phenotype (Anokhin, Grant, Mulligan, & Heath, 2015; Sanchez-Roige et al., 2018) and that it is associated with greater addiction liability in preclinical and human models (e.g. Dougherty et al., 2014; Oberlin & Grahame, 2009; Perry, Larson, German, Madden, & Carroll, 2005; VanderBroek, Acker, Palmer, de Wit, & MacKillop, 2016), suggesting that it plays a role in the development of addictive disorders. Collectively, these results suggest that DD is a promising transdiagnostic psychological mechanism (Bickel et al., 2019) and may therefore be compatible within the RDoC framework. Within the five RDoC domains, DD is a sub-construct nested in Positive Valence Systems.

However, there are limitations in the current literature regarding empirical studies testing DD as a transdiagnostic indicator. In particular, DD is typically examined in studies exclusively examining individual forms of psychopathology (e.g. individuals with alcohol use disorder compared to a control group), adjusting for pertinent covariates, such as income, but without incorporating concurrent psychopathology. Psychiatric comorbidity among conditions is well known to be high (Hasin & Grant, 2015), but there are few studies that have addressed the relationship between DD and multiple forms of psychopathology simultaneously, meaning that across this diverse literature, the potential for confounding is also high. Notably, there is evidence of shared genetic underpinnings of DD and both substance use and psychiatric conditions. Sanchez-Roige et al. (2018) found DD had a significant genetic correlation with ever smoking, daily smoking level, and successful quitting (inversely), as well as with the presence of major depressive disorder and severity of depressive symptoms, implying transdiagnostic relevance. Nonetheless, few behavioral studies have explicitly examined whether DD is transdiagnostically informative in relation to multiple conditions concurrently.

To address these issues, and to test the hypothesis that DD is a transdiagnostic construct more explicitly, the current study sought to examine these questions in a large non-treatment-seeking sample of community adults. Specifically, three complementary strategies were used to offer different vantage points. First, the study individually examined DD in relation to clinical cut-off scores for a number of common psychiatric syndromes [i.e. substance use disorders, major depressive disorder, anxiety disorder, posttraumatic stress disorder (PTSD), and ADHD], paralleling early case-control studies. Second, the study used principal components analysis (PCA) to generate aggregate indicators of psychopathology and examined DD in relation to these measures of latent overlap. Third, the study used structural equation modeling (SEM) to examine the unique relations between DD and psychopathology when simultaneously modeling multiple syndromes concurrently.

## Method

### Participants

The sample consisted of a cohort of community adults (*n* = 1388) who completed a one-time in-person assessment as part of enrollment in a health research registry at St. Joseph's Healthcare Hamilton. To be eligible, participants were required to be between the ages of 18–65 and agree to complete a one-time in-person assessment of health-related information, psychological variables, and other related information. Participants were also required to have no major or terminal medical conditions that would preclude voluntary participation in any subsequent studies.

### Measures

#### Delay discounting

DD was assessed using the Monetary Choice Questionnaire (MCQ; Kirby, Petry, & Bickel, 1999), which comprises 27 dichotomous choices between receiving a smaller monetary reward sooner, or a larger monetary reward later (e.g. ‘*Would you rather have $30 today or $80 in 30 days*’). All choices were for hypothetical monetary rewards. Hyperbolic temporal discounting functions, Mazur's (1987) *k* parameter, are inferred from participant choices at three levels of reward magnitude: small ($25–$35), medium ($55–$65), and large ($75–$85). Three control items that offered larger and smaller rewards with no delay (e.g. *Would you rather $85 today, or $55 dollars today*) were admixed among the items. These provide a quality control metric to measure low effort or attention. Participants choosing the lower monetary reward for any of these items were excluded. In addition, consistency was calculated for each of the reward magnitudes to assess the degree of correspondence between each of the responses and their inferred *k* value. Individuals who had <90% consistency were excluded.

#### Psychiatric indicators

*Alcohol Use Disorder Identification Test* (AUDIT; Saunders, Aasland, Babor, De la Fuente, & Grant, 1993). The AUDIT is a screening tool for alcohol severity. It is comprised of 10 items, with three categories, alcohol intake (items 1–3), alcohol dependence (items 4–6), and adverse consequences (items 7–10), and a total score range from 0 to 40. Participants are asked about their alcohol use within the past year. A score of 8 and above is considered the standard cut-off for hazardous drinking. The AUDIT demonstrated good internal consistency (*α* = 0.80).

*Drug Use Disorder Identification Test* (DUDIT; Berman, Bergman, Palmstierna, & Schlyter, 2005). The DUDIT is a screening tool for problematic drug use, with questions pertaining to frequency, dependency, physical and psychological issues associated with drug use excluding alcohol, cannabis, and cigarette use. It contains 11 items, with scores ranging from 0 to 44. A score of 8 and above is considered the standard cut-off for problematic drug use. The measure demonstrated excellent internal consistency (*α* = 0.91).

*Cannabis Use Disorder Identification Test Revised* (CUDIT-R; Adamson et al., 2010). The CUDIT is a measure of cannabis use frequency and severity, adapted from the AUDIT. It contains 10 items, with scores ranging from 0 to 40. A score of 6 and above for males and 2 and above for females is considered the standard cut-off for hazardous cannabis use. The measure was found to have good internal consistency (*α* = 0.78).

*Fagerstrom Test for Nicotine Dependence* (FTND; Heatherton, Kozlowski, Frecker, & Fagerstrom, 1991). The FTND is a six-item measure of the intensity of nicotine dependence, with scores ranging from 0 to 10. Scores from 1 to 2 are associated with low dependence, 3–4 associated with low to moderate dependence, 5–7 associated with moderate dependence, and 8 and above associated with high dependence. A score of 5 and above is considered the standard cut-off for problematic nicotine use. The measure demonstrated acceptable internal consistency (*α* = 0.74).

*Adult ADHD Self Report Scale* (ASRS; Kessler et al. 2005a, 2005b). The ASRS is an 18-item self-report measure of ADHD symptoms, with scores ranging from 0 to 72. A cut-off of 14 and above is used as a screen for ADHD. The measure demonstrated excellent internal consistency (*α* = 0.90).

*Patient Health Questionnaire* (PHQ-9; Spitzer, Kroenke, & Williams, 1999): The PHQ-9 is a self-report measure of depressive symptoms. It contains nine items, and each symptom is evaluated as to whether or not they occurred over the past 2 weeks. A cut-off of 10 and above is used as a screen for depression. The measure was found to have good internal consistency (*α* = 0.89).

*Patient Health Questionnaire Anxiety Subscale* (PHQ-Anx; Kroenke, Spitzer, Williams, & Löwe, 2010). The PHQ-Anx is a seven-item scale assessing anxiety symptoms. Scores range from 0 to 3 with a total score ranging from 0 to 21. Each symptom is evaluated as whether or not they occurred over the past 2 weeks. A cut-off of 10 and above is used as a screen for anxiety. The measure demonstrated good internal consistency (*α* = 0.76).

*Posttraumatic Stress Disorder Checklist-5* (PCL-5; Weathers, Litz, Herman, Huska, & Keane, 1993). The PCL-5 is a 20-item self-report measure assessing PTSD symptom severity. Symptoms are evaluated based on their occurrence over the past 2 weeks. A cut-off of 32 and above is used as a screen for PTSD. The PCL-5 as a whole demonstrated good internal consistency (*α* = 0.95).

#### Data analysis

The data were assessed for missing values, normality, and outliers. In total, four individuals were excluded based on the MCQ control items and 40 participants were excluded based on low consistency. Excluded participants constituted 3.07% of the sample. As preliminary analyses, zero-order correlations were conducted for each of the variables to examine the relationship between the MCQ, each psychiatric condition, and candidate covariates including sex, age, and income. This was to characterize unadjusted associations for health behaviors and potential nuisance variables. A three-step data analytic strategy was implemented. First, to examine the hypothesis that DD will be elevated in conditions independently, ANCOVAs of DD were conducted based on clinical cut-offs for each of the clinical indicators, adjusting for age and income and comparing individuals who screened positive to those who screened negative for a given measure. In these analyses, a single consolidated index of DD was generated via PCA using each of the three reward magnitudes. In contrast to a mean or omnibus scoring, PCA was employed as it includes the differential loading of the three magnitudes on the aggregate DD variable, providing somewhat greater resolution, and it is more similar to the latent variable approach in the SEM analyses. Conceptually, these analyses addressed the extent to which DD was elevated when multiple forms of psychopathology were examined concurrently, albeit independently. Second, a PCA of the psychopathology variables was conducted (direct oblimin rotation) to identify latent aggregations of the psychiatric syndromes and partial correlations (adjusting for income and age), and subsequently the components were examined in relation to the PCA-derived DD variable. Conceptually, this analysis was to examine the extent to which DD was associated with observed forms of overlapping psychopathology. Third, a structural equation model was used to explore the association between DD and each psychiatric condition when modeled together simultaneously using Mplus (Muthén and Muthén, 1998–2012). A single latent variable was created from each of the reward magnitudes on the MCQ (i.e. $30, $55, and $80), in accordance with previous literature that suggests an inverse association between the rates of discounting and the delayed reward magnitude (Kirby, 1997), as well as studies reporting significant differences across all three reward magnitudes on the MCQ (Kirby & Maraković, 1996; Kirby et al., 1999). As such, modeling DD using three reward magnitudes instead of simply using a mean value provides the opportunity to characterize the loadings of the specific magnitudes for the latent indicator. In particular, SEM was selected as it can explore the simultaneous unique associations between each of the disorders and the latent construct of DD, while controlling for the correlations among the dependent variables. In addition, SEM also has the ability to explicitly assess measurement error, estimate a latent construct that is not observable in the data, and can generate a structure and assess the fit of the data to that structure. Conceptually, this analysis was intended to examine the specificity of associations among DD and other health behaviors. The model was examined using the established criteria for the four model fit indices: Comparative Fit Index (CFI) >0.90 (Ullman, 2001), Tucker–Lewis Index (TLI) >0.95 (Hu & Bentler, 1999), the Root Mean Square Error of Approximation (RMSEA) <0.08 (MacCallum, Browne, & Sugawara, 1996), the root mean square residual (SRMR) <0.10 (Hu & Bentler, 1999), and chi-square test of model fit (χ^2^) (*p* > 0.05 Hu & Bentler, 1999).

## Results

### Preliminary findings

Missing data were unsystematic and rare (maximum/variable = 0.28%) and were therefore estimated using the full information maximization likelihood approach. To account for skewness, all *k* values were log_10_ transformed, which provided adequate correction. Participant characteristics are in Table 1. Frequencies of participants scoring at or above the clinical cut-off score for each disorder are also presented in Table 1. A heat map of zero-order (Pearson) correlations (*r*) is presented in Table 2; exact associations are in supplemental materials. Significant correlations were found between each reward magnitude on the MCQ and most mental health variables, with the exception of the AUDIT (all magnitudes) and the ASRS (large magnitude only). Of the candidate covariates, only age and income were significantly associated with each of the reward magnitudes, and therefore sex was dropped from the subsequent analysis. Effect sizes for significant associations between DD and symptom domains were generally small in magnitude.

**Table 1.** Participant characteristics (*N* = 1388)

Variable	%/M (s.d.)/median			
Sex (% female)	57.9			
Age	38.99 (13.70)			
Race (% white)	81.9			
Education (years)	15.34 (3.22)			
Income	⩾$75 000 to <$90 000 CAD	% above cut-off		
PHQ-Anx	3.75 (3.57)	7.6		
PHQ-9	5.07 (5.14)	15.2		
PCL-5	12.13 (13.57)	9.7		
ASRS	8.52 (4.40)	22.9	*% past-month use*	*M* (*s.d.) past-month users*
AUDIT	4.24 (4.16)	16.1	89.9%	4.57(4.14)
CUDIT	2.18 (4.73)	11.3	29.2%	3.71(5.69)
DUDIT	1.27 (4.04)	12.6	12.9%	7.72(7.86)
FTND	0.52 (1.53)	7.5	22.0%	1.01(2.02)

**Table 2.** Zero-order correlations among variables

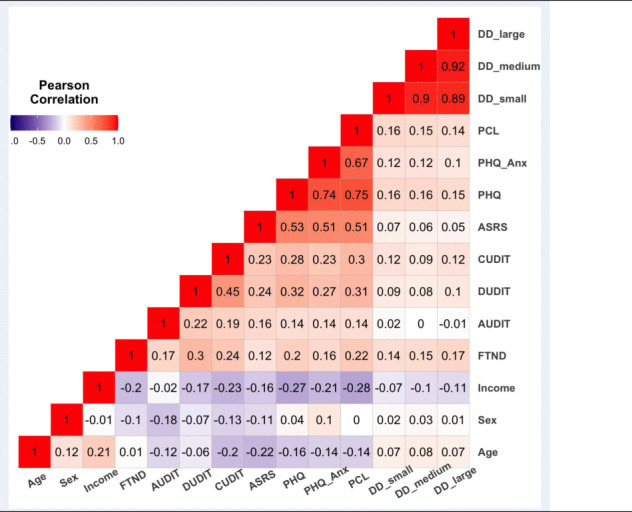

FTND, Fagerstrom Test for Nicotine Dependence; PHQ, Patient Health Questionnaire-9; PHQ-Anx, Patient Heath Questionnaire Anxiety Scale; PCL, Posttraumatic Stress Disorder Checklist-5; AUDIT, Alcohol Use Identification Test; CUDIT, Cannabis Use Identification Test; DUDIT, Drug Use Identification Test; ASRS, Adult ADHD Self Report Scale; DD, delay discounting.

### Individual syndrome comparisons

**Fig. 1.**
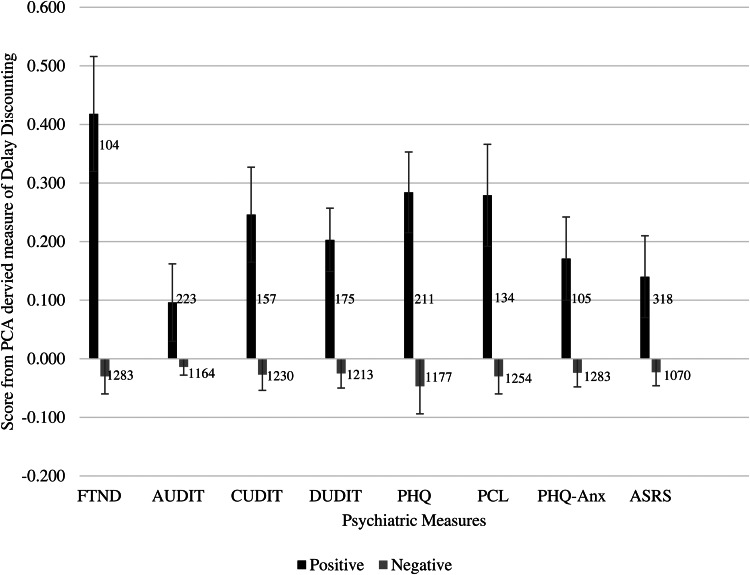
Estimated marginal means (±SEM) of PCA-derived levels of delay discounting by clinical cut-off for each domain. Numbers reflect the *n*s screening positive or negative. *Note*: FTND, Fagerstrom Test for Nicotine Dependence; PHQ, Patient Health Questionnaire-9; PHQ-Anx, Patient Heath Questionnaire Anxiety Scale; PCL, Posttraumatic Stress Disorder Checklist-5; AUDIT, Alcohol Use Identification Test; CUDIT, Cannabis Use Identification Test; DUDIT, Drug Use Identification Test; ASRS, Adult ADHD Self Report Scale.

Significant results from the ANCOVAs are presented in Fig. 1. Significant elevations in DD were present for tobacco use disorder (*F* = 19.35, *p* < 0.001), cannabis misuse (*F* = 10.07 *p* = 0.002), drug use disorder (*F* = 7.88, *p* = 0.005), depression (*F* = 19.22, *p* < 0.001), PTSD (*F* = 12.65, *p* < 0.001), anxiety (*F* = 4.04, *p* = 0.045), and ADHD (*F* = 6.36, *p* = 0.012). The effect sizes were all small, ranging from 0.003 for ADHD and anxiety to 0.014 for tobacco use disorder and depression. Of note, DD was not significantly elevated in individuals screening positive for alcohol misuse.

### Principal components analysis of psychiatric indicators

Two components were identified using the PCA oblimin rotation analysis, accounting for 58.58% of the total variance. The first component significantly contributed to explaining the relationship between depression, anxiety, PTSD, and ADHD; and the second component significantly contributed to explaining the relationship between substance use disorders (see pattern matrix in Table 3). Partial correlations adjusting for income and age revealed significant positive relationships between the first component and PCA-derived DD variable (*r* = 0.150, *p* < 0.001), and the second component and DD (*r* = 0.140, *p* < 0.001).

**Table 3.** Pattern matrix from principal component analysis of each psychiatric indictor

Variable	Component	
	1	2
PHQ-Anx	0.892	−0.051
PHQ-9	0.896	0.014
PCL-5	0.851	0.056
ASRS	0.730	0.002
AUDIT	−0.053	0.557
CUDIT	0.080	0.691
DUDIT	0.091	0.730
FTND	−0.045	0.657
% of variance	42.22	16.36

FTND, Fagerstrom Test of Nicotine Dependence; PHQ-9, Patient Health Questionnaire-9 (depressive symptoms); PHQ-Anx, Patient Heath Questionnaire Anxiety Scale; PCL-5, Posttraumatic Stress Disorder Checklist-5; AUDIT, Alcohol Use Identification Test; CUDIT, Cannabis Use Identification Test; DUDIT, Drug Use Identification Test; ASRS, Adult ADHD Self Report Scale

### Structural equation modeling evaluation

**Fig. 2.**
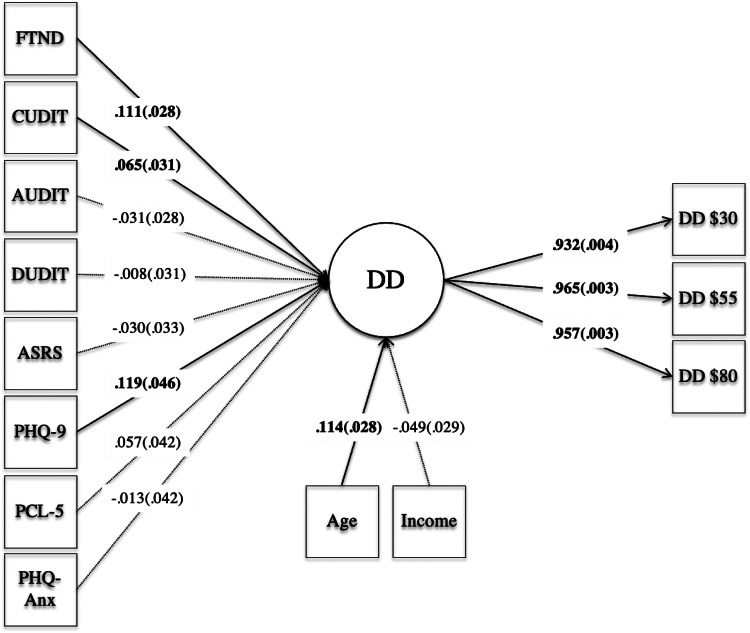
Model of a latent variable of delay discounting at three reward magnitudes in relation to dimensional indicators of substance use, attention-deficit hyperactivity disorder, depression, anxiety, and posttraumatic stress disorder. *Notes*: Solid lines and bolded values indicate significant loadings, and dotted lines indicate non-significant loadings. FTND, Fagerstrom Test for Nicotine Dependence; PHQ-9, Patient Health Questionnaire-9 (depressive symptoms); PHQ-Anx, Patient Heath Questionnaire Anxiety Scale; PCL-5, Posttraumatic Stress Disorder Checklist-5; AUDIT, Alcohol Use Identification Test; CUDIT, Cannabis Use Identification Test; DUDIT, Drug Use Identification Test; ASRS, Adult ADHD Self Report Scale; DD, delay discounting.

The model revealed an excellent model fit for each of the fit indices, CFI = 0.996, TLI = 0.993, RMSEA = 0.029, and SRMR = 0.005. Of note, χ^2^ = 42.711 (*p* = 0.002, df = 20), however this value is highly sensitive to sample sizes above 400 and may not be as interpretable as other fit indices (Saris, Satorra, & Van der Veld, 2009). Standardized model results are in Fig. 2; coefficients with 95% confidence intervals are in online Supplementary materials. As expected, all three magnitudes for the MCQ loaded well on the single latent variable (standardized coefficients >0.90). Significant positive associations were observed between the latent variable of DD and severity of tobacco dependence (FTND), cannabis misuse (CUDIT), and depression (PHQ-9). Alcohol misuse (AUDIT), illicit drug use (DUDIT), anxiety (PHQ-Anx), PTSD (PCL-5), and ADHD (ASRS) were not significantly associated with DD in the concurrent model. With regard to covariates, age, but not income, was positively associated with impulsive DD.

## Discussion

The current study sought to evaluate DD as a transdiagnostic RDoC indicator by examining its relationship to a number of psychiatric domains independently and concurrently. Elevated DD was observed for individuals scoring above the clinical cut-off for tobacco use disorder, cannabis misuse, drug use disorder, depression, PTSD, anxiety, and ADHD. In concurrent examinations, using PCA, DD was significantly associated with a two-component structure reflecting substance use disorders and all other mental disorders, which may suggest some transdiagnostic potential for DD across disorders. However, using SEM, a more precise measurement approach, the results implicate DD in tobacco use, cannabis use, and depression, but not other substance use, ADHD, anxiety, or PTSD, thus not supporting DD as a transdiagnostic process across the majority of conditions. Notably, there were associations across forms of psychopathology, in some cases of large magnitude. This is not surprising given epidemiological studies documenting high rates of comorbidity (Conway, Compton, Stinson, & Grant, 2006; Kessler et al., 2005a, 2005b), but as these behaviors and symptoms are ‘fellow travelers’, the possibility of confounding is present. Further, in our subsequent analysis exploring the relationships between DD and each psychiatric condition separately, the results suggest that exclusively examining DD in terms of individual relationships with specific forms of psychopathology may not be accounting for key variables, such as smoking status or level of depression, and thus included a third variable confound in which the observed link was in fact attributable to an unobserved variable (e.g. smoking). Repeated instances of inadvertent confounding would spuriously imply transdiagnostic relevance at the higher level of the literature. This is of course conjecture, but the facts of the current results fundamentally indicate some specificity in the links between DD and psychiatric domains, not a fully domain-general transdiagnostic relationship.

Among the significant associations in the combined analysis, the most robust association was with smoking, which may be because the very nature of cigarette smoking recapitulates DD itself (i.e. many small episodes of using cigarettes at the cost of future outcomes). For example, a pack-a-day smoker is engaging in 20 decisions a day for the smaller-sooner reward. In addition, studies have suggested when combining tobacco use with other substances, smoking accounts for a significant portion of the variance in DD. For example, studies have suggested that individuals with an SUD such as alcohol use disorder or cocaine use disorder discount substantially more when combined with heavy smoking, as compared to drinking or cocaine use alone (García-Rodríguez, Secades-Villa, Weidberg, & Yoon, 2013; Moody, Franck, Hatz, & Bickel, 2016). While many studies have explored the relationship between smoking alone and DD, most of the literature on DD does not explore smoking simultaneously with other SUDs. As such, it may be that once smoking is included, the association between other substances naturally attenuates. Interestingly, DD was also significantly associated with the severity of cannabis use. This association has been less frequently observed in the literature compared to other substances and studies that have examined DD and cannabis use have found inconsistent results, with some finding increased DD is associated with cannabis use (e.g. Sofis, Budney, Stanger, Knapp, & Borodovsky, 2020), and other studies finding no such relationship (e.g. Johnson et al., 2010). A recent meta-analysis, however, observed a small omnibus effect size between increased DD and cannabis use frequency and severity (Strickland, Lee, Vandrey, & Johnson, 2020), suggesting an aggregate association. Here, it was notable that cannabis use was most highly correlated with illicit drug use, which is common, and previous studies on DD and illicit drugs have not (to our knowledge) adjusted for cannabis use, thus potentially introducing a confound.

A surprising aspect of the current results was that the substance use associations were specific to smoking and cannabis in the SEM analyses, and non-significant for alcohol and illicit drugs, although the absence of associations is not without precedent. Individual studies have reported null findings for alcohol (e.g. MacKillop, Mattson, Anderson MacKillop, Castelda, & Donovick 2007; Moody et al., 2016), for example. In addition, most substance use studies have focused on a single drug at a time and have also not systematically cataloged and adjusted for other substances that were not the focus of the study. Thus, the relationship between DD and substance use may be more nuanced than typically thought. In support of this, a recent study using the Addiction Neuroclinical Assessment (ANA) approach to deep-phenotyping heavy drinking found that DD loaded into the executive dysfunction domain (Nieto et al., in press) and that domain was associated with a family history of alcoholism but not drinking itself.

Amongst the other mental health conditions, DD was specifically associated with depression in the SEM analyses. One explanation for this finding is higher DD may be related to a specific feature of depression, either a symptom or a cluster of symptoms or behaviors. For example, Pulcu et al. (2014) found increased hopelessness in individuals with depression was significantly associated with elevated DD. In other words, individuals who have a negative, bleak outlook on the future may experience an increased tendency to discount the value of a larger reward later. However, it is important to note that not all studies confirmed these findings. For example, Lempert and Pizzagalli (2010) observed decreased DD in individuals who displayed greater anhedonia. In addition, it is also important to note that depression was highly correlated with other psychopathology in the sample, such as anxiety and PTSD. Screening tools for these conditions tend to commonly measure negative affectivity and are thus not orthogonal from one another. Nonetheless, among these three domains, the results clearly implicate depressive symptoms as being uniquely associated with DD.

Another notable finding is the positive association between DD and age. There have been conflicting accounts in the literature regarding the relationship of DD and age (Green, Fry, & Myerson, 1994; Read & Read, 2004); however, these studies typically do not explore conditions simultaneously or use discrete age bands rather than a continuous variable. The results from this study suggest that age may be a significant factor when accounting for multiple conditions, but further research is needed to confirm this association.

This study's findings should be considered in the context of its strengths and limitations. First, this study utilized validated dimensional screening instruments, but not formal diagnostic tools. As a result, performance on these measures does not reflect a definitive clinical diagnosis. Moreover, while these tools are widely used, it may be the case that other measures may be more sensitive. Second, this was a cross-sectional study and therefore no conclusions can be made about the temporal ordering of the relationship between DD and psychopathology in these findings. Third, this study did not examine the full breadth of psychopathology, such as personality disorders, eating disorders, or psychotic disorders. However, the study did utilize a large sample size, providing high statistical power and increasing the generalizability of the findings. In addition, the study examined participants who were non-clinical community adults, again increasing generalizability. However, as this is a non-clinical sample, the level of severity of symptoms is substantively lower than clinical samples. As such, it could be the case that differences in DD become more pronounced at higher levels of severity. For these reasons, the current findings should not be considered definitive, but illustrative of the need for more investigation of DD in the context of multiple domains of psychopathology.

At a broader level, another consideration is that DD as an indicator is a form of revealed preference that may in fact reflect multiple underlying mechanisms. That is, multiple conditions may be associated with the phenotype of elevated DD, but for different reasons. For example, it may be that high DD is variably a result of lower executive control, higher reward processing, present-focused ruminative cognition, or hopelessness about the future. Triangulating potentially different behavioral or neurobiological mechanisms is an important future direction. Taken together, there is a high need for future DD studies that are carefully designed to test RDoC hypotheses.

In sum, the findings from this study suggest that the prospects for DD as a transdiagnostic indicator may be more complex than conjectured. Exploring DD independently suggests associations with a large number of psychiatric domains, but concurrently examining DD in relation to multiple psychiatric conditions reveals more limited linkages. The findings reveal some transdiagnostic significance in concurrent models (for smoking, cannabis use, and depression), but no evidence in relation to a broad swath of psychiatric domains. More broadly, this study emphasizes the need for additional methodologically rigorous and careful study designs investigating DD and other transdiagnostic RDoC indicators.

## Supplementary material

For supplementary material accompanying this paper visit https://doi.org/10.1017/S0033291721005110. click here to view supplementary material
