# Supplementary material for: Evaluation of delay discounting as a transdiagnostic research domain criteria indicator in 1388 general community adults
Source: Psychol Med. 2022 Jan 26;53(4):1649–57. doi: 10.1017/S0033291721005110 (PMC10009385; doi:10.1017/S0033291721005110)
Supplement: Supplementary file 1 [file S0033291721005110sup001.docx]

**Evaluation of Delay Discounting as a Transdiagnostic Research Domain Criteria Indicator in General Community Adults**

**SUPPLEMENTARY MATERIALS**

Figure S1. Zero-order correlations among variables.

| Variable | 1. | 2. | 3. | 4. | 5. | 6. | 7. | 8. | 9. | 10. | 11. | 12. | 13. |  |
| --- | --- | --- | --- | --- | --- | --- | --- | --- | --- | --- | --- | --- | --- | --- |
| 1. Age | - | - | - | - | - | - | - | - | - | - | - | - | - |  |
| 2. Sex | .122** | - | - | - | - | - | - | - | - | - | - | - | - |  |
| 3. Income | .210** | -.011 | - | - | - | - | - | - | - | - | - | - | - |  |
| 4. FTND | .013 | -.095** | -.199** | - | - | - | - | - | - | - | - | - | - |  |
| 5. CUDIT | -.195** | -.126** | -.230** | .241** | - | - | - | - | - | - | - | - | - |  |
| 6. AUDIT | -.119** | -.185** | -.022 | .165** | .188** | - | - | - | - | - | - | - | - |  |
| 7. DUDIT | -.058* | -.071* | -.174** | .301** | .447** | .218** | - | - | - | - | - | - | - |  |
| 8. ASRS | -.222** | -.112** | -.156** | .117** | .234** | .159** | .238** | - | - | - | - | - | - |  |
| 9. PHQ-9 | -.162** | .039 | -.274** | .199** | .281** | .135** | .316** | .528** | - | - | - | - | - |  |
| 10. PCL-5 | -.145** | -.003 | -.276** | .223** | .305** | .139** | .309** | .513** | .746** | - | - | - | - |  |
| 11. PHQ-Anx | -.136** | .096** | -.211** | .158** | .228** | .138** | .266** | .510** | .745** | .673** | - | - | - |  |
| 12. DD $30 | .072** | .017 | -.069* | .144** | .122** | .022 | .094** | .066* | .161** | .156** | .116** | - | - |  |
| 13. DD $55 | .076** | .032 | -.101** | .147** | .090** | -.001 | .081* | .058* | .161** | .150** | .124** | .900** | - |  |
| 14. DD $80 | .074** | .015 | -.106** | .166** | .116** | -.006 | .098** | .048 | .150** | .143** | .100** | .892** | .923** |  |
| Note: FTND: Fagerstrom Test for Nicotine Dependence; PHQ-9, Patient Health Questionnaire-9; PHQ-Anx, Patient Heath Questionnaire Anxiety Scale; PCL-5, Posttraumatic Stress Disorder Checlist-5; AUDIT, Alcohol Use Identification Test; CUDIT, Cannabis Use Identification Test; DUDIT, Drug Use Identification Test; ASRS, Adult ADHD Self Report Scale; DD, delay discounting | | | | | | | | | | | | | | |

**Table S1. Standardized Model Results**

| Variable | β | SE | *p* | CI |
| --- | --- | --- | --- | --- |
| FTND | 0.111 | 0.028 | <.001 | [0.055, 0.166] |
| CUDIT | 0.065 | 0.031 | 0.037 | [0.004, 0.125] |
| AUDIT | -0.031 | 0.028 | 0.263 | [-0.085, 0.023] |
| DUDIT | -0.008 | 0.031 | 0.796 | [-0.069, 0.053] |
| ASRS | -0.030 | 0.033 | 0.361 | [-0.094, 0.034] |
| PHQ-9 | 0.119 | 0.046 | 0.010 | [0.028, 0.211] |
| PHQ-Anx | -0.013 | 0.042 | 0.750 | [-0.095, 0.068] |
| PCL-5 | 0.057 | 0.042 | 0.181 | [-0.026, 0.140] |
| Age | 0.114 | 0.028 | <.001 | [0.059, 0.169] |
| Income | -0.049 | 0.029 | 0.090 | [-0.105, 0.008] |
| Note: FTND: Fagerstrom Test for Nicotine Dependence; PHQ-9, Patient Health Questionnaire-9; PHQ-Anx, Patient Heath Questionnaire Anxiety Scale; PCL-5, Posttraumatic Stress Disorder Checklist-5; AUDIT, Alcohol Use Identification Test; CUDIT, Cannabis Use Identification Test; DUDIT, Drug Use Identification Test; ASRS, Adult ADHD Self Report Scale. | | | | |
